# Preparation of Cosmetic Emulsions Containing *Hippophae* Oil Isolated by Various Methods: Study of Their Antioxidant, Sun-Blocking and Physicochemical Properties

**DOI:** 10.3390/antiox12101829

**Published:** 2023-10-04

**Authors:** Sofia S. Zosimidou, Evangelia C. Vouvoudi, Ioannis S. Tsagkalias, Smaro S. Lykidou, Nikolaos F. Nikolaidis

**Affiliations:** Laboratory of Polymers and Dyes Chemistry and Technology, Department of Chemistry, Aristotle University of Thessaloniki, GR-54124 Thessaloniki, Greece

**Keywords:** oil extraction, carotenoids, natural ingredients, sea buckthorn, *Hippophae rhamnoides* L., cosmetology, pyrolysis, fatty acids

## Abstract

An industry listed as one of the largest globally is the cosmetic industry. In recent years, this industry has shown growing interest in the application of natural ingredients providing advanced properties to cosmetic creams such as moisturizing, antioxidant, sun-protecting and antimicrobial effects. In this context, the present study concerns the production of cosmetic emulsions containing *hippophae* oil obtained via the methods of extraction, hydro-distillation and maceration using sunflower oil as the carrier oil. Firstly, an IR-ATR analysis was performed showing that the oils prepared were close to those commercially obtained. Then, the stability of the emulsions was tested over a time period of four months through measuring their pH and viscosity values with positive outcomes, and their antioxidant ability was also measured using the DPPH method. The latter one showed that *hippophae* oil greatly improves the antioxidant capacity. Moreover, based on the fact that sea buckthorn contains carotenoids, the SPF value of the emulsions was determined. The results showed that the addition of *hippophae* oil to the emulsions gave higher absorption in UV-Vis, thus higher SPF values. Py-GC/MS analysis was used to identify decomposition compounds in the produced oils. Among those, valuable compounds such as *Ω*-6, *Ω*-7 and *Ω*-9 fatty acids and many aldehydes were found by the decomposition of the oils.

## 1. Introduction

A cosmetic product (term of Greek origin meaning the one that ornaments) defines every substance that intends to come into contact with the external parts of the human body, with the main purpose of cleaning, protecting, perfuming or changing their appearance [1]. Cosmetic creams are products whose viscosity does not allow them to flow at room temperature, usually emulsions or gels. Emulsions, depending on the nature of the dispersed phase, are distinguished as oil in water *o/w* or water in oil *w/o* or both.

“Cosmeceuticals” is an innovative term describing the cosmetic products that demonstrate some pharmaceutical activity [2,3], usually by containing natural ingredients of beneficial results. North America is a major market for natural and organic personal care products, followed by Europe and Asia–Pacific, where China and India play a significant role in the global herbal cosmetics market. Natural skin care maintains top billing in the global organic beauty market and is expected to emerge as the most attractive segment with a 30.9% share by 2024 [2]. The market of herbal beauty products may reach US$ 73 billion in 2023, since the target market nearly holds a 5.9% share in the overall beauty products industry [4]. The global consumption of plant-based cosmetics has shown spectacular growth in recent years, thanks to social media penetration which raised consumers’ awareness of the beneficial contribution of the traditionally used, naturally based ingredients. The numbers reveal the peoples’ interest and choice over the natural cosmetics. The multidisciplinary cooperation of researchers is crucial to analyze their functional properties and effectiveness [1].

Essential oils, called such because they are believed to comprise the quintessence of scents and flavors from the floral/plant kingdom, bear a very complicated synthesis, usually unknown in detail. They are volatile at ambient conditions, with a characteristic aroma, mostly colorless substances, isolated from an aromatic plant by a natural process. They can be found in various parts of the plant such as the fruit, leaves, roots, flowers, barks, seeds and resins [1]. In chemistry terms, such oils are terpenes (mainly monoterpenes and sesquiterpenes) [5], phenolics and alcohols, i.e., oxygenated derivatives of terpene hydrocarbons such as aldehydes, ketones, alcohols, phenols, acids, ethers and esters [6]. Their quality and chemical composition varies not only among plant types, but also according to the stage of growth of the plant, the part of the plant from which they were extracted, the time of day, the season of the year and the climate of the country in which the plant thrives or is cultivated [7,8,9,10,11]. To maintain better cosmetic and healing properties, essential oils must be pure, unadulterated and cultivated in ideal conditions. Their storage conditions also play an important role since they are very sensitive.

The most common methods used for the industrial extraction of these oils are steam distillation, solvent extraction, pressing, and hydrolysis, as well as new methods in which ultrasounds and microwaves are applied. The choice depends on the characteristics of the material from which the oil will be extracted, as it can be present in different parts of the plant, such as the roots, stem, leaves, fruit and/or seeds.

Sea buckthorn (*Hippophae rhamnoides* L.) is a thorny shrub widely distributed in Asia, some regions of Europe (including Greece) and China [12]. It grows orange fruit (berries) with a unique composition of bioactive components that are usually found only separately. Fruits and leaves have been used in the past for medical purposes, such as eliminating phlegm, improving blood circulation or hepatoprotection [12,13], especially in China. The bioactive components vary with fruit maturity, fruit size, species, geographic location, climate and methods of extraction [13]. *Hippophae* berries are rich in vitamins C, E and P, as well as malic acid and citric acid. They mainly contain vitamin C, but also vitamin A, i.e., *α*- and *β*-carotene and other carotenoids. In addition, the berries contain tocopherols, i.e., vitamin E, folic acid and vitamin B complex group [13]. The fruit contains flavonoids, catechins and procyanidins, cyclitols, phospholipids, tannins, sugars, organic acids (maleic acid, oxalic acid, malic acid, tartaric acid), and phenolic acids [13,14], as well as fatty oils (in berries’ pulp up to 8% wt and in seeds up to 12.5% wt). The content of vitamin C depends on the variety of the plant and its geographical location. For example, the sea buckthorn that grows in Europe on coastal dunes contains 120–315 mg% of vitamin C in fresh fruit, while the species that grows in the Alps contains much more vitamin 405—1100 mg% [15]. When the berries are pressed, the resulting juice separates into three layers: the upper layer is a thick orange cream, the middle layer contains a mixture of saturated and unsaturated fatty acids, and the lower layer is a juice, a source of fat used for cosmetics [15].

During the isolation process, overheating of the raw material should be avoided to avoid the decomposition of various components of the essential oil [16]. In the case of distillation in water, the hydrolysis of esters and the difficulty of taking all O-compounds from the aquatic phase are probable. Extraction is the process that most frequently gives more enriched products; although the method is relatively simple and quite efficient, it suffers from disadvantages such as long extraction time, relatively high solvent consumption and often unsatisfactory reproducibility [17]. The use of ultrasound waves increases the pressure and results in penetration and transport effects, while the increase in temperature accelerates diffusion and dissolution effects. With the use of ultrasounds, the extraction time is reduced, smaller volumes of solvents are used and many substances are extracted at the same time [16].

There are several in vitro methods for the determination of the antioxidant activity of an ingredient, mainly based on the ability of the antioxidant substance to donate *e*^−^ to H atoms [18]. One of these methods is the “DPPH method”, which is based on the interaction of a stable chemical radical, 2,2-diphenyl-1-picrylhydrazyl, with an antioxidant, which results in the discoloration and reduction of the absorption of the radical [19,20]. This method was developed by Blois [21], with a view to determining the antioxidant activity by using a stable free radical 2,2-diphenyl-1-picrylhydrazyl (DPPH). It is a decolorization method (purple to yellow) that measures the ability of antioxidants to react directly with the DPPH radical by measuring the UV-Vis absorbance at 517 nm using a UV-Vis spectrophotometer [22]. Scientific research based on kinetic analysis between phenols and DPPH proved that the reaction behaves as an electron transfer reaction [23]. The DPPH method is a rapid, simple, inexpensive and widely used method that can be used for solid and liquid samples of food or biological origins. Its disadvantage is that it is only used for the determination of all antioxidants and is not limited to the measurement of a specific component [24]. This is essential for natural ingredients or plant extracts because they are always a mixture of certain substances that act as antioxidants. DPPH reactions are extremely sensitive to the conditions of the system where the reaction takes place, i.e., the presence of water and solvent, pH, oxygen and light exposure. The stable radical DPPH interacts with active molecules and is reduced through the addition of an H-atom (hydrogen atom transfer) and converted into 2,2-diphenyl-1-picrylhydrazine, which has a yellow color, as a result of which absorbance at 517 nm is reduced [24].

The emulsion stability refers to its resistance to changes in its properties over time; thus for *o/w* systems, this is achieved by choosing an emulsifier that is more soluble in the aqueous phase. Changes in the stability of an emulsion can be caused by various physicochemical processes or by the influence of microbiological factors. Various substances are found in common cosmetic emulsions for pharmaceutical reasons, but also as molecules necessary for their physicochemical stability. In cosmetics, xanthan gum is used as an emulsion stabilizer, as a surface-active substance, and as a binding agent, as it causes an increase in the viscosity of the product. It gives a richer appearance and a thicker texture, and at the same time allows them to become slightly runny after shaking. Similarly, stearic acid is used as a surface-active substance that helps to stabilize the emulsion by allowing the mixing of the oily phase with the aqueous phase. Polysorbate-60 acts as an emulsifier, stabilizing the emulsion and facilitating the solubility of substances in solvents in which they could not previously be dissolved. In any case, before applying a recipe, the “EC Directive on Cosmetics” and the “Cosmetic Ingredient Review” [25,26] provide regulations to determine what is considered safe for use in cosmetics.

The aim of this study is to isolate *hippophae* oil from *hippophae* berries provided commercially via various isolation methods. Cosmetic creams for skin care were prepared using the *hippophae* oils extracted, and their antioxidant, sunscreen and some physicochemical properties were investigated over time.

## 2. Materials and Methods

### 2.1. Materials and Oil Isolation Methods

**Figure 1:**
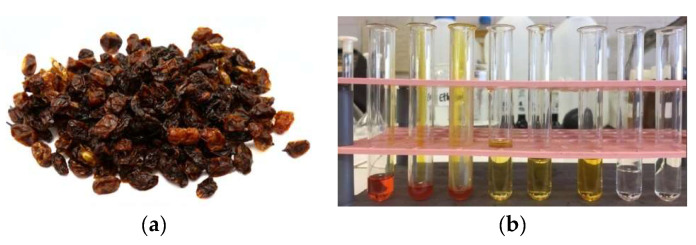
(**a**) The dried berries from which the oil was isolated, (**b**) The 8 oils applied in emulsions (from left to right): the commercial Siberian oil, oil extracted in EtOH, oil extracted in hexane, the commercial Greek oil, oil isolated with cold maceration, oil isolated with hot maceration, oil after distillation in H_2_O, sunflower oil.

Six different treating methods have been established in order to isolate *hippophae* oil from *hippophae* dried berries (Figure 1a), commercially obtained by a local herbal market (Avramoglou, Thessaloniki, Greece). The berries were dried (30 min, 50 °C) for moisture loss and grounded in a household mill. Apart from the oils isolated using laboratory techniques, two commercially available *hippophae* oils were purchased, one of Greek origin from a local herbal market (Avramoglou, Thessaloniki, Greece) and one of Siberian origin (Aveo, Russia), and applied in the emulsions as well. Regarding the berries, the isolation processes were:

(a) Cold maceration: 10 g of dried and ground *hippophae* berries were added in closed vessel with 100 g sunflower oil (edible grade, commercial). The container was shaken every 2 days and placed in a refrigerator at 4 °C for 14 days. The solids were then filtered and the oil phase was collected.
(b) Hot maceration: 10 g of dried and ground *hippophae* berries were added in closed vessel with 100 g sunflower oil (edible grade, commercial). The mixture was placed in a water bath at a temperature of 60 °C for 3 h. The solids were then filtered and the oil phase was collected.
(c) Distillation in H_2_O: 40 g of dried and ground *hippophae* berries were added in a 500 mL spherical flask, along with deionized H_2_O up to 2/3 of its volume. A vertical insert and a side condenser were attached for the collection of the distillate. The heat was controlled so ca. 20 drops/min were collected. When the H_2_O volume lowered in the flask, small quantities were added from the upper opening of the Claisen adapter. In total, 200 mL of distillate was collected after 3–4 h. When the drops were blurred, the presence of oil was evident in water, when the drops were transparent, no more oil could be extracted. The distillate was kept for a day in ambient dark conditions in a ground glass stopped flask. Next, in a separatory extraction funnel of 250 mL, 20 mL of diethylether (Riedel-de-Häen, Germany) was transferred along with the distillate, so the oil migrated to the organic phase, which was the upper phase in the liquid–liquid extraction. The aquatic phase was then washed in diethylether again to collect the total oil. Into the 50 mL product (diethylether with oil) some molecular desiccant was added to absorb the water traces for 1 h. The diethylether mix was heated in mild conditions at 40 °C in a water bath for the ether evaporation. Caution was taken for the temperature not to rise rapidly and no usage of a hot plate because the ether is flammable.
(d) Extraction in H_2_O: 20 g of dried and ground *hippophae* berries were added in a 250 mL conical flask, along with 200 mL of deionized H_2_O. The flask was placed in a water bath at 60 °C and left for 3 h. Then it was filtered and the liquid water phase was collected.
(e) Extraction in EtOH: 20 g of dried and ground *hippophae* berries were added in a 250 mL conical flask, along with 200 mL of EtOH (>98%, Merck, Germany). Sonication was performed for 30 min (Grant XUBA 3) followed by filtration in a Buchner funnel with paper Whatman No 1, 110 mm in order to separate the liquid. The solvent was evaporated in water bath at 70 °C, and the oil was isolated.
(f) Extraction in hexane: 20 g of dried and ground *hippophae* berries were added in a 250 mL conical flask, along with 200 mL of hexane (Merck). Sonication for 30 min followed by pressure filtration in a Buchner funnel with paper Whatman No 1, 110 mm to isolate the liquid. The solvent was evaporated in water bath at 60 °C, thus the oil was isolated.

The isolated oils were placed in a closed dark container with a ground glass stopper and stored in a cool, dry and shady place (for up to 12 months). In the absence of light, as long as no color change or odor change was observed, the oils were considered excellent for further use (Figure 1b).

### 2.2. Emulsion Preparation

The emulsions prepared were of the *o/w* type and involved three stages: preparation of the aqueous phase, preparation of the oily phase, and mixing of the two phases for the formation of an emulsion (balance EMB 200-2, Kern ± 0.01 g).

(a) Aqueous phase (75% of emulsion) consists of deionized water (70%), glycerin (3.5%), xanthan gum (1%) and tetrasodium EDTA (0.5%). In the case of the cream containing aqueous extract of *hippophae* oil, the water was replaced by the extract in the recipe. Similarly, in the case of oil distilled in water, the distillate replaced the water in the recipe.
(b) Organic phase (23%) was prepared in a beaker with the *hippophae* oil or the sunflower oil (11%), cetylstearyl alcohol (2%), cetyl alcohol (2%), polysorbate-60 (2%), stearic acid (2%), shea butter (2%) and finally the beeswax (2%).
(c) The two beakers were placed in the water bath of 80 °C for homogenization. Then, the oil phase was added to the aqueous phase gradually, under stirring at 100 rpm (RW 14 H, Janke and Kunkel Ika Werk, Staufen, Germany) while temperature was kept constant at 80 °C. When the addition was completed, heating was terminated while the mixture remained under stirring in the water bath. The total stirring ranged from 1.5–2 h until complete formation of the emulsion (cream) was observed. Due to the fact that phenoxyethanol (preservative, 1%) and ethylhexylglycerin (1%) are volatile components, their addition was made at a temperature below 50 °C. Afterwards, the cream was transferred into plastic containers and kept in a dark, cool place for storage.

FDA lists all above ingredients as safe and approved ingredients for cosmetic applications, as does the Cosmetic Ingredient Review. Each oil isolated was used for the preparation of the corresponding cream, while an additional cream was also prepared as a reference sample, using just sunflower oil and no *hippophae* oil. In total, 9 creams were prepared (200 g each) and used throughout this research work.

### 2.3. Characterization of Emulsions

For the stability of the emulsions prepared, pH (WTW 535, Gemini BV, Haaksbergen, The Netherlands) and viscosity measurements (Visco Star plus, Fungilab SA, Barcelona, Spain) were executed at certain time intervals after their preparation: 1 day, 1 and 2 weeks, 1, 2 and 3 months. For the viscosity measurements, after testing various spindles, the use of spindle R3 was chosen, and was used for the viscosity measurements after being immersed to a certain height in the containers.

For the determination of the sun protection factor (SPF index), an in vitro spectroscopical method was applied: solutions of 1% *w*/*v* cream in EtOH (Scharlau, Barcelona, Spain) were prepared with the aid of a sonication processor (UP 100H). The solution was further diluted with EtOH and 2 mL of this solution became 10 mL of a new solution. A Shimadzu UV-1800 Spectrophotometer equipped with UVProbe ver. 2.61 software (Shimadzu, Kyoto, Japan) was used for obtaining the absorption spectra with D-lamp. The optical cells used were 1 cm width Starnaglass cells (type 1, material G, Hainault Industrial Estate, Ilford, UK). The reference sample was the pure EtOH solvent. The use of the Mansur equation is necessary for the calculation of SPF index spectrophotometrically [27]:

(1) $$\mathrm{SPF}=CF\times \sum_{290}^{320}EE\left(\lambda \right)\times Ι\left(\lambda \right)\times A\left(\lambda \right)\;$$

where *ΕΕ*(*λ*) is the erythemal effect of the spectrum at wavelength λ, *Ι*(*λ*) is the solar intensity of the spectrum at wavelength *λ*, *CF* is the correction factor equal to 10, *A*(*λ*) is the absorption in 290–320 nm region (every 5 nm), while the values for the term “*EE* × *I*” are normalized constants determined by Sayre et al. [28].

The antioxidant properties of the emulsions were determined using the DPPH method: the 2,2-diphenyl-1-picrylhydrazyl (Lot# STBH7297, Sigma Aldrich^®^, Burlington, MA, USA) solution was prepared at 50 mg/L in EtOH while the cream solutions were prepared at 1 %*w*/*v* concentration in EtOH too. Then 1 mL of cream solution was mixed with 3 mL of DPPH solution and sonication followed for 30 min. The solutions prepared were very transparent, no particles in suspension, no turbidity noticed. The absence of light is obligatorily in the whole process, so the flasks were covered with aluminum foil. A blank solution was also prepared with 1 mL of pure EtOH mixed with 3 mL of DPPH solution. The absorbances were recorded on the UV-Vis (W-lamp working) where recordings were in the visible range with λ_max_ = 517 nm. A reduction in the absorbance of the mixtures indicated a higher DPPH scavenging activity. The equation elaborated is:

(2) $$\mathrm{Free}\;\mathrm{radicals}\;\mathrm{scavenging}\;\mathrm{activity}\;(\%)=\frac{A_{C}-A_{S}}{A_{C}}\;$$

where *A_S_* and *A_C_* represent the absorbance of each sample and of the control sample (no cream), respectively. The stock solution of DPPH slowly deteriorates so it may be used for small period of time [24].

The Perkin-Elmer Spectrum One IR Spectrophotometer (USA) was used for the identification of the functional groups of the oils isolated. The Spectrum v.5.3.1 (2004) software and the necessary auxiliary HATR equipment were elaborated. The spectra of the 8 oil samples were taken in the range 4000–700 cm^−1^, resolution 4 cm^−1^ and number of scans 32. The total reflectance occurred on a ZnSe plate and 45°.

Pyrolysis experiments were carried out at a QP2010 Ultra GC/MS (Shimadzu, Japan) equipped with a Multi-Shot Pyrolyzer EGA/PY-3030D (Frontier, Japan) under inert conditions of flowing He. The single-shot pyrolyses were executed after various trials and tests occurred for the method development, on a Mega 5HT capillary column (Italy) of 30 m × 0.25 mm × 0.25 μm geometry, non-polar in nature, covered with 95% dimethylsiloxane. Eventually, the set of parameters and conditions analyzed below were determined. Pyrolysis at 250 °C occurred for 0.2 min, while interfaces’ temperatures were at 280 °C. The GC column oven remained at 40 °C for 1 min, heated until 220 °C at 4 °C/min and then held for 4 min (50 min of elution). The column flow set at 1 mL/min, the purge flow of He at 3 mL/min and the total flow at 104.1 mL/min had, as a result, the pressure at 49.5 kPa, while linear velocity yield was at 36.1 cm/s. Several trials regarding the split and the column flow led to ratio 100, to avoid saturation. The MS parameters involved the ion source stable at 200 °C, the detector voltage at 1.2 kV, the *m*/*z* range at 50–600 and the scan speed at 10,000. A tiny drop of each liquid was required, transferred in cups (stainless steel, coated with molten SiO_2_). The evaluation of the results was carried out through GC/MS Lab Solutions, v.2.71 (Shimandzu, Kyoto, Japan 2011) and “Search analysis program & Data” (NIST11 ver.1.00, Shimandzu, 2011, Japan).

## 3. Results

This study presents the successful isolation of *hippophae* oil from dried sea buckthorn berries with various extractions, and the preparation of *o*/*w* emulsions (creams) including the isolated oils as active ingredients. Moreover, it examines the main properties of the oils and the emulsions to investigate whether the beneficial properties are transferred by the active ingredient to the cream.

**Figure 2:**
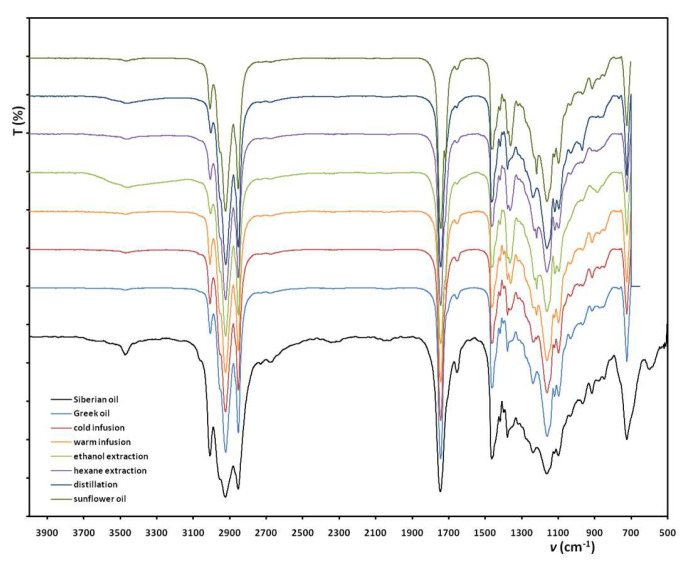
IR spectra of the oils studied, both obtained commercially and isolated experimentally.

Figure 2 illustrates the ATR spectra of oils collected experimentally along with the two commercial oils obtained. The primary observation is that the spectra are similar and many identical peaks are recorded in all of them. The baseline of the spectra is excellent and the peaks are well-shaped; thus the technique applied for the IR recordings was efficient. Examining the spectra from left to right we may identify the following absorptions: at 3474 cm^−1^ the –OH stress vibration, 3009 cm^−1^ corresponds to the =C-H bonds; at 2922–2934 cm^−1^ the –CH_2_– and –CH_3_ groups symmetrically stressed and at 2854 cm^−1^ asymmetrically stressed; at 1744 cm^−1^ the great C=O bond; at 1656 cm^−1^ the absorption for C=C bonds; at 1464 cm^−1^ the –CH_2_– bonds scissoring absorptions; at 1418 cm^−1^ the C-H absorption; at 1378 cm^−1^ the –CH_2_– bending; at 1163 cm^−1^ the C-O absorption; and while in the fingerprint area the peak at 915 cm^−1^ is for the C=C and at 723 cm^−1^ for the –CH_2_– rocking band vibrations [29]. Regarding the fingerprint area, since the samples studied are natural ingredients, it may be considered a larger part of the spectra *pe.* than the 1800–700 cm^−1^ region. The number, shape and absorptions of the peaks in the fingerprint area may indicate subtle differences, such as the variety of the plant or the part of the plant.

Regarding the spectra shown in Figure 2, it may be stated that all samples prepared experimentally are identical in terms of peak intensity too, meaning that the isolation techniques chosen where appropriate for the extraction of the essential oil from the dried fruit. Moreover, they were executed properly in detail, so that the desired oil was eventually isolated in pure form (identical with the commercial one) and the solvents that interfered were removed satisfactorily. As for the *hippophae* oil of Siberian origin, it was in a denser form; this is why its IR peaks are a bit wider, but still at the same absorption wavelengths. The characteristic groups mentioned correspond to some categories of organic compounds often found in natural ingredients; these are the fatty acids (presence of –OH and C=O) and the carotenoids, where only C=C bonds and –CH_3_ may be found, while esters are also possible. In fact, 1743 cm^−1^ implies a high presence of carotenoid esters, but C=O group is present in volatile oils, triglycerides, and aliphatic esters found in the extracts as well. A peak at 1463 cm^−1^ , apart from carotene, can also be assigned to lycopene pigments [29]. Thus, we observe the presence of terpenes and unsaturated hydrocarbons along with some esters. The peaks at 915 cm^−1^ (*trans* -CH=CH-) are attributed to *β*-carotene. Certainly, the existing literature informs us that other categories of compounds are also evident in sea buckthorn oil, such as tocopherols or sterols [3], yet their characteristic groups are of the same kind as noticed above.

**Figure 3:**
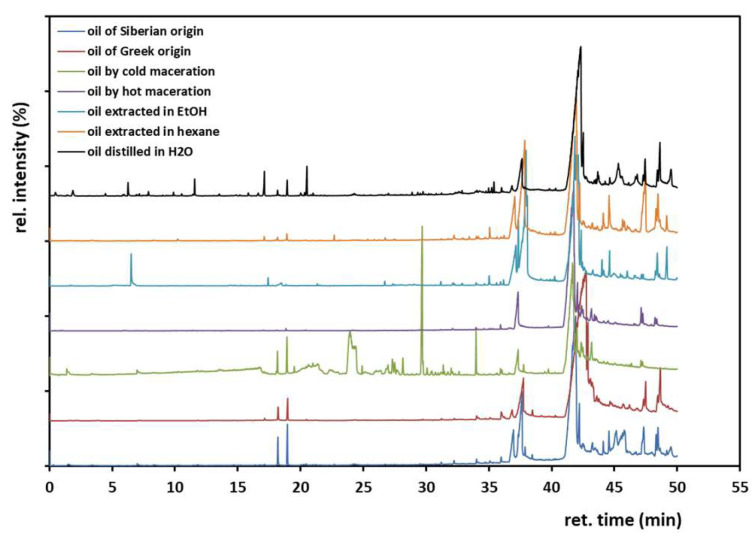
The chromatograms of the fragments eluted in GC/MS when the oils were pyrolyzed at 250 °C.

GC/MS analysis of the *hippophae* oils followed. The chromatograms are shown in Figure 3, and the fragments yielded by the oils pyrolyzed at 250 °C are summarized in Table 1. The method was applied after investigating the parameters of oven temperature, column flow and split ratio mainly. The elution method developed managed to satisfactorily separate the products yielded, as shown by the sharp peaks drawn and the flat baseline. The adopted elution method allowed the main fragments to be separated in 50 min, where the last 15 min included the majority of the eluted molecules (yet, not numerous peaks). The chromatograms seen in Figure 3 were very helpful for ingredients determination. It must be noticed that no solvent peaks were recorded in the beginning of the chromatograms; thus all solvents experimentally elaborated for the isolation processes have been eventually successfully removed. Peaks similar in shape and size are shown among the chromatograms of the oils examined after the 35th min of elution, with some greater or medium peaks, whose repetition at standard retention times demonstrates the identical chemistry of the initial samples. The compounds identified in the oils examined are shown in Table 1, all found through the NIST11 library, with a similarity percentage of over 70%. Since they are nature-based ingredients, we are not surprised by the stereochemistry that was noticed for the compounds, yet this is extremely hard to verify.

Oleic acid is a monounsaturated *Ω*-9 fatty acid identified in all samples; palmitoleic acid is an *Ω*-7 monounsaturated fatty acid found too. Pentadecanoic acid, hexadecanoic acid (palmitic acid) and 9,12-octadecadienoic acid, which is an *Ω*-6 fatty acid, are also among the findings. This was anticipated since sea buckthorn berries are rich in fatty acids in the range of 12–22 carbon atoms, usually in *cis* configuration [3].

The compound eluted at t = 17–18 min of the elution is (*E,E*)-2,4-decadienal, a substance used for aroma, which at low concentrations, has the odor of citrus fruits. In the case of the oil isolated by hydro-distillation, we noticed some smaller peaks in the range of 6–22 min, compared to the other chromatograms. Those peaks for the *hippophae* isolated with water distillation correspond to aldehydes, such as hexanal, heptanal, octanal, and decanal, all found naturally in citrus fruits. On the other hand, derivatives of ascorbic acid and aldehydes of C_16_-C_18_ structures are derived along with some sterols and benzoates. As shown in Table 1, most products found are O-compounds, yet some alkenes were detected too, like 1-tridecene, 9-tricosene, (*Z*)-pentacosane, tetracontane, nonacosane or squalene. All are in accordance with some of the 100–200 ingredients that exist in *hippophae* fruit, as stated in the literature [15].

**Figure 4:**
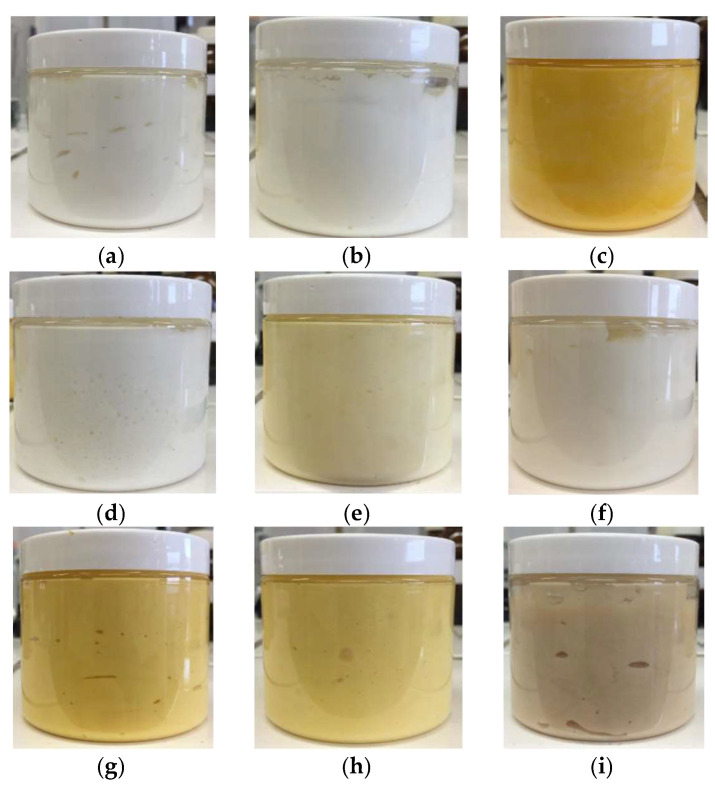
Images of the emulsions prepared: (**a**) reference cream, (**b**) cream with commercial oil from Greek market, (**c**) cream with commercial oil of Siberian origin, (**d**) cream with oil from cold maceration, (**e**) cream with oil from hot maceration, (**f**) cream with oil distilled in H_2_O, (**g**) cream with oil extracted in hexane, (**h**) cream with oil extracted in EtOH and (**i**) cream with extraction in H_2_O.

In Figure 4, images of the emulsions prepared using the oils obtained are demonstrated, right after preparation. The intense yellow color is evident for the cream containing the commercial thick oil of Siberian origin (Figure 1b), and the creams prepared with oil extracted in hexane, EtOH. The aquatic distillation of the oil and the hot maceration of the berries give a beige hue in the emulsions, compared to the total white reference cream (sunflower oil only). The oil extraction in H_2_O does not produce an oily phase, but a mixture of polar compounds extracted from the berries, so as it was mentioned, this extraction replaced the aquatic phase in the emulsion. The product is not recorded in ATR and Py-GC/MS (Figure 2 and Figure 3) because of the great water peaks that do not help with the identification of the rest of the groups or fragments.

**Figure 5:**
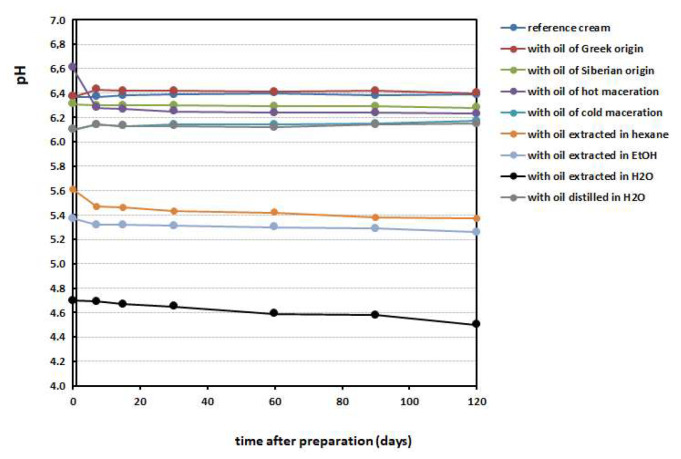
pH values recorded for the emulsions prepared, impressively stable in a 4-month time period.

Figure 5 shows the changes in pH values measured for all of the creams over time, from the first day until the fourth month after their preparation. The stability of the pH values over 4 months is impressive, showing the endurance of the recipe followed. Regarding the direct application of the prepared creams onto skin, we may conclude that the creams containing the oils isolated through extraction in EtOH and hexane provide the friendly pH character (5.5 for human skin). The other creams need some modification after being isolated or the cream recipe requires modification for pH adjustment before use (pH = 6–6.6).

**Figure 6:**
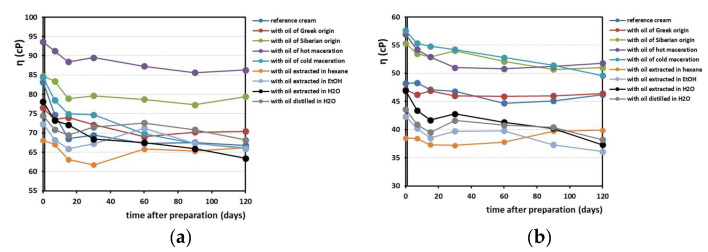
Trends of viscosity values of the emulsions prepared in a 4-month time period, measured with stirrer at (**a**) 50 rpm and (**b**) 100 rpm measurements.

Figure 6 shows the viscosity changes recorded over a period of 4 months in the creams prepared. The trend of the viscosity values is a slight decreasing, and this is reasonable to occur over time. We also noticed that the 50 rpm and 100 rpm stirrers provoked different results in viscosity range. The values obtained begin at 65–95 cP for the 50 rpm stirrer and 35–60 cP for the 100 rpm stirrer, and their drop occurs after 2 weeks.

**Figure 7:**
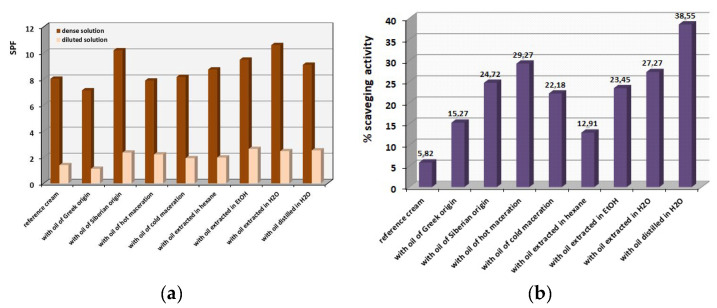
(**a**) SPF values, indicating the sunscreen protection, and (**b**) free radicals scavenging activity percentages, indicating the antioxidant activity, calculated using UV data and Equations (1) and (2).

Figure 7a shows the SPF values calculated using Equation (1) from the UV absorbances as described in the experimental section. There are two groups of calculations, one for the diluted samples, and the other for the concentrated samples prepared by the same emulsions. As could be anticipated, the concentrated samples provided higher SPF values than the diluted ones, since they are richer in the sun-blocking active ingredients. The extra sunscreen protection of almost all emulsions seems to be higher compared to the reference cream where no *hippophae* oil is contained. The higher SPF protection of 11 units was afforded by the creams containing the Siberian oil and the aqueous extract vs. the almost SPF 8 of the reference cream with no added *hippophae* oil. Thus, the oils may need to be added in higher percentages in order to afford higher sunscreen protection. However, one should have expected high sun-blocking efficiency from *hippophae* cosmetic creams due to the presence of many repeatable single and double carbon bonds in the same molecules which can absorb a high level of UV irradiation.

Finally, Figure 7b demonstrates the scavenging activity towards the free radicals applying the DPPH method, detected for the emulsions prepared. The results are truly impressive, since the scavenging activity towards the free radicals increased significantly: two to seven times compared to the reference cream that did not contain any *hippophae* oil. The antioxidant values are shown in Figure 7b. It can be concluded that the isolation methods that involve thermal treatment, such as hot maceration and distillation, provide the greater scavenging of free radicals. It can be concluded that the *hippophae* oil provided exceptional antioxidant activity in all cosmetic preparations performed. This can be attributed not only to the abundant presence of fatty aliphatic acids (e.g., octadenanoic, hexadecenoic, oleic etc.) or fatty aldehydes (e.g., decadienal, hexadecenal, etc.), but to the ascorbic acid (vitamin C) and carotenoids well known for their conferred antioxidant properties. The *hippophae* oil-containing cosmetic creams provide an abundant source of free radical scavengers due to the presence of suitable molecules able to trap free radicals.

## 4. Discussion

In the present work, cosmetic creams of *o/w* type emulsions containing *hippophae* oil (sea buckthorn oil) were prepared using various methods, such as the maceration with carrier oil, water distillation or extraction with organic solvents. In addition, the stability of the emulsions was checked with measurements of pH and viscosity, measurements of the sunscreen protection index in vitro and the study of the antioxidant properties of the emulsions after the IR and GC/MS analysis of the oils isolated.

From the visual results, we conclude that the emulsions containing the commercial *hippophae* oil of Siberian origin and those produced with oils obtained by extraction in organic solvents show a more intense shade of orange compared to others which appear slightly yellow-white. The pH measurements made in relation to time had good repeatability. The rheological properties of emulsions can be controlled in many ways, such as by an increase in the dispersed phase or emulsifier, or even the selection of a more viscous continuous phase [30]. Literature comparisons are rare to occur among investigations of emulsions, since each investigation applies various concentrations of extracts added and various temperature and humidity keeping conditions for periods of time. The general saying is that the presence of EtOH lowers the viscosity of the emulsions over time [30]. The total removal of EtOH or hexane in *hippophae* oils prepared in this study helped with the stability of the viscosity of the *o/w* emulsions kept at ambient conditions for 4 months. For more complicated emulsions with higher aquatic content and various natural ingredients, a beforehand mixture design used as an optimization tool is considered a good idea [30].

Evaluation of the efficiency of the sunscreen protection of a product has, for a long time, been assessed using in vivo tests, performed on human volunteers. Yet, the in vitro tests using laboratory measurements have shown remarkable reliability, convenience and consistency in numerous cases, and indicate the actual sunscreen capability of the formulations [31]. In order to develop products with high SPF, the formulators must understand the physicochemical principle, and consider not only the UV absorbance of the active ingredients, but also the “vehicle” components, such as esters, emollients and emulsifiers used in the emulsions, since sun-blocking substances can interact with other components, and these interactions can affect sunscreens efficacy [27]. From the values obtained for the sunscreen protection index, we concluded that the emulsions showed a slightly higher in vitro sun protection index compared to the reference emulsion (sunflower oil only, no *hippophae* ingredient). In addition, the emulsion containing the oil by the hot maceration method also showed a lower SPF index calculated than the reference sample. This is probably due to the fact that some precious components may have broken down, due to the heating of the oil during the application of the method. The capability of long-chain polyene structures to physically quench electronically excited molecules is well known in Chemistry. Likewise, carotenoids, the major class of polyene compounds in biology, which occur as important plant pigments, have been studied extensively. Since carotenoids surround humans as part of their normal diet and nature, interest has extended to nutrition and medicine [32]. Carotenoids have been shown to maintain the stability of oils against photo-oxidation [33] and generally have antioxidant action that protects the skin from sunburn caused by UV_B_ radiation. The exposure to solar UV radiation has been estimated to be ~10% for outdoor-working adults and ~3% for indoor-working adults of the total available annual UV radiation. The high UV absorption recorded in Figure 7a is due to carotenoids; they have a large chromophore group and conjugation of double bonds with the carboxyl groups, which can create a system capable of absorbing high energy. The excitation energy is transferred to the carotenoid, which, in turn, dissipates the excitation energy to the solvent (as heat) and returns to the initial ground state. Thus, a repeating cycle is generated, so that the carotenoids can undergo another round of singlet oxygen quenching [32].

Antioxidants such as vitamins (vitamin C, vitamin E), flavonoids, and phenolic acids play the main role in fighting against free radical species, which occur in many reactions due to various causes. Effective botanical antioxidant compounds are widely used in traditional pharmacopoeia and include tocopherols, flavonoids, phenolic acids, Ν-containing compounds and monoterpenes. Vitamin C (ascorbic acid) is not only good for skin conditioning (against acute UV_B_ damage), but also is considered good for the development of herbal cosmetic formulation that can diminish the probability of skin cancer and the photoaging process. *Hippophae* oil compared to vitamin C shows greater antioxidant capacity [12]. Vitamin E, a free radical scavenger and an emollient too, also presents protective health activity like decreasing immunosuppression, photoageing, erythema and photo carcinogenesis [34].

Antioxidant DPPH measurements showed that the presence of sea buckthorn oil enhances the antioxidant activity of the emulsions up to seven times, a truly huge improvement, compared to the reference emulsion. Greater antioxidant capacity was given by the emulsion with aqueous-distilled oil, a result probable due to the greater purity of the distillate (fewer ingredients are taken away at narrow temperature range). In addition, the hot maceration method also showed satisfactory results as the increased temperature favored the quality of the oil. In all cases, emulsions showed greater antioxidant capacity than the reference cream. The typical composition of sunflower oil is palmitic acid 5%, stearic acid 6%, oleic acid 30%, linoleic acid 59% *v*/*v*. Flavonoids are the main antioxidant components of *Hippophae rhamnoides* L., influenced by the heat and the oxidation stress [12]. Sanwal et al. showed that the DPPH activity of sea buckthorn oil varies in value, affected by treatment time, exposed temperature, ultrasound power and solvent to sample ratio; thus our results are also influenced by the method of isolation [16]. The application of lower temperatures or shorter elution times in combination with ultrasound treatments results in high antioxidant content in the oils. The various technologies used in the food industry are not entirely favorable to active compounds contained in plants. For example, phenolic compounds can be degraded by high temperatures [14]. However, research applying similar experimental techniques to sea buckthorn berries of Indian origin reached antioxidant activity of 26% [13], close to our results regarding hot and cold maceration and extraction in H_2_O and EtOH. On the other hand, no significant differences in antioxidant activity of the extracts from regular and roasted seeds were noticed, in regard to lipid peroxidation [14]. The usage of dried berries in this research did not deprive us of any of the useful, active, rich ingredients (as SPF and free radicals scavenging results proved), but on the contrary, saved us from concerns with handling the fresh fruit.

Finally, gas chromatography/mass spectrometry showed the existence of substances such as *Ω*-6, *Ω*-7 or *Ω*-9 fatty acids in the analyzed oils. Oleic, palmitic and palmitoleic acids are the fatty acids found in all oils prepared and in various analyses in literature, especially in *hippophae* pulp and fruit of European origin [13]. Methylesters of oleic, stearic, myristic, linoleic or palmitic acids are a major part of elutants in conventional GC/MS [16]. Volatile ingredients that are possibly released at short treatment exposures include aldehydes (aroma ingredients), but various literature also includes some ketones and benzoates [12]. Palmitoleic acid (*Ω*-7) is a rare fatty acid, a component of skin lipids, that stimulates regenerative processes in the epidermis and promotes wound healing [3]. Apart from their beneficial properties in human nutrition, all these are components of frequent use in the cosmetics industry due to the moisturizing, antioxidant [35], antiaging [12,36], antimicrobial [37], anticancer [38] and anti-inflammatory properties [15] they offer.

A considerable factor for the quality of the oil used and the content transferred in the emulsions is the origin of the parts of the plant used. Slawinska et al. showed that the fruit, branches, and leaves vary in quantities, and the fresh fruits provide higher quality compared to the roasted ones [14]. In terms of antioxidant capacity it is reported that a leaf > stem > fruit order exists for *Hippophae rhamnoides* L. [12]. In contrast to fatty acids and carotenoids, the activity of polysaccharides or flavonoids is harder to be evaluated.

## 5. Conclusions

The evaluation of the active ingredients of the *hippophae* oil isolated from dried *hippophae* berries of Greek origin is the aim of this study. The target product for including that essential oil is a skin care cream with advanced antioxidant capacity. As it was proven, the isolation of the essential oil is possible via a maceration process (hot and cold), an extraction process (with polar and non-polar solvent) and via distillation in aqua. All of these methods succeeded in producing oils with enhanced antioxidant activity since the emulsions prepared increased the ability to scavenge free radicals by three to seven times. As Py-GC/MS analysis proved, a variety of *Ω*-3, *Ω*-6, *Ω*-7, *Ω*-9 fatty acids are included in all oils retrieved, along with ascorbic acid and valuable esters. The emulsion prepared showed stable behavior in terms of pH and viscosity values during a period of 4 months. The route for the inclusion of functional ingredients such as the natural essential oils of valuable plants is multidimensional, and it is worth undertaking more research. It has been verified that due to the well-balanced content of fatty acids, carotenoids and vitamins, *hippophae* oil may be incorporated in “cosmeceuticals” because it retards skin aging, absorbs harmful irradiation, and protects skin against inflammations and bacteria growth.

## Figures and Tables

**Table 1:** The fragments identified in GC/MS (ret. time 27–45 min) when pyrolyzing the oils at 250 °C.

Sample	Compound	Formula
Greek origin	(*E,E*)-2,4 –Decadienal	C_10_H_16_O
	1-(+)- Ascorbic acid 2,6-dihexadecanoate	C_38_H_68_O_8_
	*n*-Hexadecanoic acid	C_16_H_32_O_2_
	oleic acid	C_18_H_24_O_8_
	Hexadecanoic acid, 1-(hydroxymethyl)-1,2-ethanediyl ester	C_35_H_68_O_5_
	9-Octadecenoic acid, 1, 2, 3-propanetriyl ester (E, E, E)	C_57_H_104_O_6_
Siberian Origin	(*E, E*)-2,4-Decadienal	C_10_H_16_O
	*cis*-9-Hexadecanoicacid	C_16_H_30_O_2_
	1-(+)- Ascorbic acid 2,6-dihexadecanoate	C_38_H_68_O_8_
	(*Z, Z*)-9,12-octadecadienoicacid	C_18_H_32_O_2_
	Pentadecanoic acid	C_15_H_30_O_2_
	Oleic acid	C_18_H_34_O_2_
	7-hexadecenal	C_16_H_30_O
	12-methyl–*E,E*-2,13-octadecadien-1-ol	C_19_H_36_O
	9,12-octadecadienoic acid	C_10_H_16_O
	17-Pentatriacontene	C_10_H_16_O
Extraction in hexane	2,4-Decadienal	C_10_H_16_O
	1-Butanol, 3-methyl-, benzoate	C_12_H_16_O
	1-Hexadecanol	C_16_H_34_O
	Oleic acid	C_18_H_34_O_2_
	Palmitoleic acid	C_16_H_30_O_2_
	1 -(+)- Ascorbicacid 2,6-dihexadecanoate	C_38_H_68_O_8_
	*cis*-9 Hexadecenal	C_16_H_30_O
	9-Octadecenoicacid, (*E, E, E*)-1, 2, 3-Propanetriylester	C_57_H_104_O_6_
	2-methylhexacosane	C_27_H_56_
Extraction in EtOH	3-octanone	C_8_H_16_O
	Butanedienoic acid, hydroxyldiethylester	C_8_H_14_O_5_
	Tetradecanoic acid, ethyl ester	C_16_H_32_O
	Palmitoleic acid	C_16_H_30_O_2_
	*n*-hexadecanoic acid	C_16_H_32_O_2_
	1 -(+)- Ascorbic acid 2,6-dihexadecanoate	C_38_H_68_O_8_
	9,12-octadecadienoic acid	C_18_H_32_O_2_
	Oleic acid	C_18_H_34_O_2_
	7-Hexadecenal	C_16_H_30_O
	Octadecanoic acid	C_18_H_36_O_2_
	Tetracosane	C_24_H_50_
Hot maceration	Octadecanoic acid	C_18_H_36_O_2_
	*n*-hexadecanoicacid	C_16_H_32_O_2_
	Oleic acid	C_18_H_34_O_2_
	*Z,Z*-8,10-Hexadecadien-1-ol	C_16_H_30_O
	Isopropyl linoleate	C_21_H3_8_O_2_
Cold maceration	(*E,E*)-2,4 Decadienal	C_10_H_16_O
	*n*-Hexadecanoic acid	C_16_H_32_O_2_
	*cis*-Vaccenic acid	C_18_H_34_O_2_
	Ascorbic acid 2,6-dihexadecanoate	C_38_H_68_O_8_
	*(Z,Z)*-9,12-octadecadienoic acid	C_18_H_32_O_2_
Distillation in H_2_O	Hexanal/Heptanal/Octanal/Nonanal	C_6–9_H_12–18_O
	Oleic acid	C_18_H_34_O_2_
	Palmitoleic acid	C_16_H_30_O_2_
	(*E, E*)-2,4-Decadienal	C_10_H_16_O
	*cis*-9-Hexadecenal	C_16_H_30_O
	7-Hexadecenal	C_16_H_30_O

## Data Availability

Not applicable.

## References

[1] Bijauliya R.K., Alok S., Kumar M., Chanchal D.K., Yadav S. (2017). A comprehensive review on herbal cosmetics. J. Pharm. Sci. Res..

[2] Nadeeshani Dilhara Gamage D.G., Dharmadasa R.M., Chandana Abeysinghe D., Saman Wijesekara R.G., Prathapasinghe G.A., Someya T. (2022). Global perspective of plant-based cosmetic industry and possible contribution of Sri Lanka to the development of herbal cosmetics. Evid. Based Complement. Altern. Med..

[3] Koskovac M., Cupara S., Kipic M., Barjaktarevic A., Milovanovic O., Kojicic K., Markovic M. (2017). Sea Buckthorn Oil—A Valuable Source for Cosmeceuticals. Cosmetics.

[4] Herbal Beauty Market. https://www.futuremarketinsights.com/reports/herbal-beauty-products-market.

[5] Lucchesi M.E., Chemat F., Smadja J. (2004). An original solvent free microwave extraction of essential oils from spices. Flavour Fragr. J..

[6] Bakkali F., Averbeck S., Averbeck D., Idaomar M. (2008). Biological effects of essential oils—A review. Food Chem. Toxicol..

[7] Figueiredo A.C., Barroso J.G., Pedro L.G., Scheffer J.J. (2008). Factors affecting secondary metabolite production in plants: Volatile components and essential oils. Flavour Fragr. J..

[8] Brooker M.I.H., Kleinig D.A. (2006). Field Guide to Eucalypts.

[9] Goodger J.Q., Heskes A.M., King D.J., Gleadow R.M., Woodrow I.E. (2008). Micropropagation of *Eucalyptus polybractea* selected for key essential oil traits. Funct. Plant Biol..

[10] Manika N., Chanotiya C.S., Negi M.P.S., Bagchi G.D. (2013). Copious shoots as a potential source for the production of essential oil in *Eucalyptus globulus*. Ind. Crops Prod..

[11] Verma R.S., Rahman L., Verma R.K., Chauhan A., Yadav A.K., Singh A. (2010). Essential oil composition of menthol mint (*Mentha arvensis*) and peppermint (*Mentha piperita*) cultivars at different stages of plant growth from Kumaon region of Western Himalaya. Int. J. Med. Aromat..

[12] Ma Q.G., He N.X., Huang H.L., Fu X.M., Zhang Z.L., Shu J.C., Wang Q.-Y., Chen J., Wu G., Zhu M.-N. (2023). *Hippophae rhamnoides* L.: A Comprehensive Review on the Botany, Traditional Uses, Phytonutrients, Health Benefits, Quality Markers, and Applications. J. Agric. Food Chem..

[13] Nazir F., Salim R., Bashir M. (2017). Chemical and antioxidant properties of Sea buckthorn (*Hippophae rhamnoides*). J. Pharm. Innov..

[14] Sławinska N., Zuchowski J., Stochmal A., Olas B. (2023). Extract from Sea Buckthorn Seeds—A Phytochemical, Antioxidant, and Hemostasis Study; Effect of Thermal Processing on Its Chemical Content and Biological Activity In Vitro. Nutrients.

[15] Zielińska A., Nowak I. (2017). Abundance of active ingredients in sea-buckthorn oil. Lipids Health Dis..

[16] Sanwal N., Mishra S., Sahu J.K., Naik S.N. (2022). Effect of ultrasound-assisted extraction on efficiency, antioxidant activity, and physicochemical properties of sea buckthorn (*Hippophae salicipholia*) seed oil. LWT Food Sci. Technol..

[17] Dawidowicz A.L., Rado E., Wianowska D., Mardarowicz M., Gawdzik J. (2008). Application of PLE for the determination of essential oil components from *Thymus vulgaris* L.. Talanta.

[18] Prior R.L., Wu X., Schaich K. (2005). Standardized methods for the determination of antioxidant capacity and phenolics in foods and dietary supplements. J. Agric. Food Chem..

[19] Williams R.J., Spencer J.P., Rice-Evans C. (2004). Flavonoids: Antioxidants or signaling molecules?. Free Radic. Biol. Med..

[20] Molyneux P. (2004). The use of the stable free radical diphenylpicrylhydrazyl (DPPH) for estimating antioxidant activity. Songklanakarin J. Sci. Technol..

[21] Blois M.S. (1958). Antioxidant determinations by the use of a stable free radical. Nature.

[22] Yu L., Zhao M., Yang B., Zhao Q., Jiang Y. (2007). Phenolics from hull of *Garcinia mangostana* fruit and their antioxidant activities. Food Chem..

[23] Huang D., Ou B., Prior R.L. (2005). The chemistry behind antioxidant capacity assays. J. Agric. Food Chem..

[24] Kedare S.B., Singh R.P. (2011). Genesis and development of DPPH method of antioxidant assay. J. Food Sci. Technol..

[25] Cosmetic Ingredient Review. https://www.cir-safety.org/ingredients.

[26] Regulation (EC) N° 1223/2009. https://single-market-economy.ec.europa.eu/sectors/cosmetics/legislation_en.

[27] Dutra E.A., Oliveira D.A.G.D.C., Kedor-Hackmann E.R.M., Santoro M.I.R.M. (2004). Determination of sun protection factor (SPF) of sunscreens by ultraviolet spectrophotometry. Rev. Bras. Ciências Farmacêuticas.

[28] Sayre R.M., Agin P.P., LeVee G.J., Marlowe E. (1979). A Comparison of In Vivo and In Vitro Testing of Sunscreening Formulas. Photochem. Photobiol..

[29] Raluca M.P., Buzoiznu A.D., Ioan V.R., Socaciu C. (2014). Untargeted Metabolomics for Sea Buckthorn. Not. Bot. Horti Agrobot..

[30] Cizauskaite U., Marksiene R., Viliene V., Gruzauskas R., Bernatoniene J. (2017). New strategy of multiple emulsion formation based on the interactions between polymeric emulsifier and natural ingredients. Colloids Surf. A Physicochem. Eng. Asp..

[31] Sheu M.T., Lin C.W., Huang M.C., Shen C.H., Ho H.O. (2003). Correlation of in vivo and in vitro measurements of sun protection factor. J. Food Drug Anal..

[32] Stahl W., Sies H. (2012). *β*-Carotene and other carotenoids in protection from sunlight. Am. J. Clin. Nutr..

[33] Jung M.Y., Min D.B. (1992). Effects of oxidized *α*-, *γ*-and *δ*-tocopherols on the oxidative stability of purified soybean oil. Food Chem..

[34] Mansuri R., Diwan A., Kumar H., Dangwal K., Yadav D. (2021). Potential of natural compounds as sunscreen agents. Pharmacogn. Rev..

[35] Olas B. (1992). Sea buckthorn as a source of important bioactive compounds in cardiovascular diseases. Food Chem. Toxicol..

[36] Sayegh M., Miglio C., Ray S. (2014). Potential cardiovascular implications of Sea Buckthorn berry consumption in humans. Int. J. Food Sci. Nutr..

[37] Suryakumar G., Gupta A. (2011). Medicinal and therapeutic potential of Sea buckthorn (*Hippophae rhamnoides* L.). J. Ethnopharmacol..

[38] Teleszko M., Wojdyło A., Rudzińska M., Oszmiański J., Golis T. (2015). Analysis of lipophilic and hydrophilic bioactive compounds content in sea buckthorn (*Hippophae rhamnoides* L.) berries. J. Agric. Food Chem..

